# Hormesis in Cholestatic Liver Disease; Preconditioning with Low Bile Acid Concentrations Protects against Bile Acid-Induced Toxicity

**DOI:** 10.1371/journal.pone.0149782

**Published:** 2016-03-07

**Authors:** Esther M. Verhaag, Manon Buist-Homan, Martijn Koehorst, Albert K. Groen, Han Moshage, Klaas Nico Faber

**Affiliations:** 1 Department of Gastroenterology and Hepatology, Center for Liver, Digestive, and Metabolic Diseases, University Medical Center Groningen, University of Groningen, Groningen, The Netherlands; 2 Department of Laboratory Medicine, Center for Liver, Digestive, and Metabolic Diseases, University Medical Center Groningen, University of Groningen, Groningen, The Netherlands; 3 Department of Pediatrics, Center for Liver, Digestive and Metabolic Diseases, University of Groningen, University Medical Center Groningen, Groningen, The Netherlands; Nihon University School of Medicine, JAPAN

## Abstract

**Introduction:**

Cholestasis is characterized by accumulation of bile acids and inflammation, causing hepatocellular damage. Still, liver damage markers are highest in acute cholestasis and drop when this condition becomes chronic, indicating that hepatocytes adapt towards the hostile environment. This may be explained by a hormetic response in hepatocytes that limits cell death during cholestasis.

**Aim:**

To investigate the mechanisms that underlie the hormetic response that protect hepatocytes against experimental cholestatic conditions.

**Methods:**

HepG2.rNtcp cells were preconditioned (24 h) with sub-apoptotic concentrations (0.1–50 μM) of various bile acids, the superoxide donor menadione, TNF-α or the Farsenoid X Receptor agonist GW4064, followed by a challenge with the apoptosis-inducing bile acid glycochenodeoxycholic acid (GCDCA; 200 μM for 4 h), menadione (50 μM, 6 h) or cytokine mixture (CM; 6 h). Levels of apoptotic and necrotic cell death, mRNA expression of the bile salt export pump (*ABCB11*) and bile acid sensors, as well as intracellular GCDCA levels were analyzed.

**Results:**

Preconditioning with the pro-apoptotic bile acids GCDCA, taurocholic acid, or the protective bile acids (tauro)ursodeoxycholic acid reduced GCDCA-induced caspase-3/7 activity in HepG2.rNtcp cells. Bile acid preconditioning did not induce significant levels of necrosis in GCDCA-challenged HepG2.rNtcp cells. In contrast, preconditioning with cholic acid, menadione or TNF-α potentiated GCDCA-induced apoptosis. GCDCA preconditioning specifically reduced GCDCA-induced cell death and not CM- or menadione-induced apoptosis. The hormetic effect of GCDCA preconditioning was concentration- and time-dependent. GCDCA-, CDCA- and GW4064- preconditioning enhanced *ABCB11* mRNA levels, but in contrast to the bile acids, GW4064 did not significantly reduce GCDCA-induced caspase-3/7 activity. The GCDCA challenge strongly increased intracellular levels of this bile acid, which was not lowered by GCDCA-preconditioning.

**Conclusions:**

Sub-toxic concentrations of bile acids in the range that occur under normal physiological conditions protect HepG2.rNtcp cells against GCDCA-induced apoptosis, which is independent of FXR-controlled changes in bile acid transport.

## Introduction

Bile acids are produced in the liver by the hepatocytes, and secreted together with water, electrolytes, cholesterol, bilirubin and phospholipids as main constituents of bile. Via the bile canaliculi and bile ducts, bile is secreted into the duodenum, aiding in absorption of dietary lipids and lipid-soluble nutrients as well as the secretion of hydrophobic toxins. [1–3] Cholic acid (CA) and chenodeoxycholic acid (CDCA) are the primary bile acids synthesized by the hepatocytes and are conjugated to the amino acids taurine or glycine, increasing the hydrophilicity of these bile acids. Unconjugated bile acids are more hydrophobic and in high dosage can damage cellular membranes through their detergent action [4,5]. Conjugated bile acids are generally more hydrophilic and can counteract the cytotoxic properties of hydrophobic primary bile acids [5]. After secretion of the bile acids in the duodenum the majority (90–95%) of the bile acids are absorbed at the terminal ileum and transported back to the liver via the portal circulation. A small portion of the primary bile acids are transformed by colonic bacteria to secondary bile acids, such as deoxycholic acid and lithocholic acid. [2,3].

In cholestatic liver disease, the bile flow from the liver to the duodenum is compromised and the enterohepatic circulation is impaired. Cholestasis can result from genetic disorders, drugs, infections, pregnancy, graft-versus-host disease and physical obstruction, such as gallstones and tumours, leading to intra- or extrahepatic blockade of bile flow and the accumulation of (cytotoxic) bile acids in the blood and liver [6,7]. The accumulation of bile acids in hepatocytes is considered to be an important pathological feature in cholestatic liver injury. At moderate concentrations, bile acids can induce apoptotic death of hepatocytes [5]. In high concentrations, bile acids function as detergents, damaging cellular membranes of the hepatocytes and inducing necrotic cell death [4,5]. Hepatocyte cell death leads to the loss of functional liver tissue and induction of a wound healing response that can progresses to liver fibrosis. Ultimately, these patients may develop liver failure [8]. Treatment options for cholestatic liver diseases are limited. In specific cases, such as primary biliary cirrhosis, primary sclerosing cholangitis and gallstone disease, the hydrophilic bile acid ursodeoxycholic acid (UDCA) is used as treatment. However, the therapeutic effect is highly variable [9,10]. Liver transplantation is the only curative treatment for advanced stages of cholestasis.

Acute cholestasis is characterized by strongly increased serum bile acid levels, inflammation and increased reactive oxygen species (ROS) production and causes massive hepatocyte death. In chronic cholestasis, inflammatory cytokines and oxidative stress generally decrease over time, while serum and hepatic bile acid levels remain high [11]. Hepatocytes adapt to the hostile environment during cholestasis, for instance by reducing the expression of the Na^+^- taurocholate cotransporting peptide (NTCP) and thereby the influx of bile acids from the blood. Simultaneously, expression of the hepatocanalicular bile salt export pump (BSEP) and basolateral multidrug resistance-associated protein (MRP) -3 and -4 is increased. BSEP enhances hepatobiliary secretion of bile acids while MRP3 and MRP4 export bile acids to the blood so that they can be cleared via the urine [2].

The nuclear receptor Farnesoid X-receptor (FXR) is a bile acid sensor and functions as an important regulator of bile acid homeostasis [12]. FXR controls bile acid production by regulating expression of *CYP7A1*, encoding the rate-limiting enzyme in primary bile acid synthesis [13]. Furthermore, FXR regulates expression of *ABCB11*, the gene encoding BSEP, as well as genes that aid in bile acid detoxification [12,14,15].

By adapting to the hostile environment, hepatocytes become resistant towards bile acid-induced apoptotic cell death, which may be explained by the principle of hormesis. Hormesis defines the adaptation of cells or organisms to a low concentration of a toxic compound. Harmful substances can have a beneficial effect in low concentrations, whereas in high concentrations the toxin can have a damaging effect [16].

The aim of this study is to investigate the mechanisms that underlie a hormetic response that protects hepatocytes against the pathological conditions in cholestasis.

We hypothesize that during the progression of cholestatic liver diseases, the increase in concentrations of bile acids, cytokines and/or ROS allow the hepatocytes to adapt to the harmful environment and increase their resistance towards apoptotic cell death.

## Materials and Methods

### Cell culture and experimental setup

Primary human hepatocytes (3 different batches) were obtained from Tebu-Bio (Heerhugowaard, the Netherlands). The HepG2-derivative cell line HepG2.rNtcp [17] was used, which is stably transfected with the rat Na^+^-taurocholate cotransporting polypeptide (Ntcp) and thereby, susceptible towards bile acid-induced cell death [5]. HepG2.rNtcp cells were cultured as described before [15]. For experimental procedures, HepG2.rNtcp cells where grown to 70% to 90% confluence in Dulbecco’s Modified Eagle Medium (DMEM, Life technologies, Breda, The Netherlands) supplemented with 100 U/ml penicillin, 100 μg/ml streptomycin, 250 ng/ml fungizone (1% PSF, Lonza, Verviers, Belgium) and 250 μg/ml geneticin (selection rNtcp expressing cells, Life technologies). The HepG2.rNtcp cells where preconditioned for 24 hr with 25 μM (sub-apoptotic concentration) of the bile acids (all obtained from Sigma-Aldrich, St. Louis, MO, USA) cholic acid (CA), glycochenodeoxycholic acid (GCDCA), taurocholic acid (TCA), tauroursodeoxycholic acid (TUDCA), chenodeoxycholic acid (CDCA), ursodeoxycholic acid (UDCA), 10 μM of the superoxide anion donor (ROS inducer) menadione (2-methyl-1,4-naphtoquinone, Sigma-Aldrich), 10 ng/ml human recombinant tumor necrosis factor alpha (TNF-α, R&D systems, Abingdon, UK), 1 μM FXR agonist GW4064 (Tocris Bioscience, Abingdon, UK), 0,1% or DMSO (control GW4064, Merck, Darmstadt, Germany). After preconditioning, the medium was refreshed and the cells were challenged with apoptotic concentrations of GCDCA 200 μM for 4 h [11], cytokine mixture (CM; containing 10 ng/ml TNF-α, 10 ng/ml IL1β, 10 ng/ml INFγ [18]), or menadione 50 μM for 6 h [19].

### Caspase-3/7 enzyme activity assay

Total cell lysates were prepared as describe earlier [5] and caspase-3/7 activity was determined with a fluorometric-based caspase-3/7 activity assay using the fluorescent caspase-3 substrate Ac-DEVD-AMC (Enzo life science, NY, USA), following manufactures protocol. Fluorescence was measured using a Bio-Tek FL600 microplate fluorescence reader (Bio-Tek, Vermont, VT, USA). The arbitrary amount of fluorescence (AU) was corrected for the amount of protein present in the cell lysates. Protein concentrations were determined using a protein assay kit (Bio-Rad, Veenendaal, The Netherlands) according to the manufacturer’s instructions. Optical density was measured with a Bio-Tek EL800 microplate reader (Bio-Tek).

### Lactate dehydrogenase release assay

The lactate dehydrogenase (LDH) release assay was used to determine necrotic cell death. After the preconditioning and challenge with bile acids, samples were taken from the cell culture medium (LDH release) and the HepG2.rNtcp cells were lysed with 100 μg/ml digitonin (Sigma-Aldrich, total LDH). LDH activity was determined by monitoring the oxidation of NADH to NAD parallel to the conversion of pyruvate to lactate [20]. The oxidation of NADH was measured at 340 nm for 30 min with 1 min interval at 37°C using a Bio-Tek EL808 Thermo microplate reader (Bio-Tek). The activity and therefore the release of LDH in the medium was calculated as percentage of the total LDH activity (LDH activity in both the medium and cell lysates).

### RNA isolation, reverse transcription PCR, and quantitative real-time PCR

Total RNA was isolated using Tri-reagent (Sigma-Aldrich), following manufacturer’s protocol. RNA quantity and quality was determined with the Nanodrop spectrophotometer (Thermo Scientific, Wilmington, DE, USA). Reverse transcription PCR (RT-PCR) was performed with 2.5 μg of RNA using the Moloney murine leukemia virus (M-MLV) reverse transcriptase system (Sigma-Aldrich), and random nanomers (Life technologies). RT-PCR was performed in 3 steps: 10 min at 25°C, 1 h at 37°C and 5 min at 95°C with the GeneAmp PCR system (Applied Biosystems, Nieuwekerk a/d IJssel, the Netherlands). Quantitative real time PCR (qPCR) was performed using 4 μl of 4-fold (*ABCB11*) or 20-fold (*NR1H4*, *NR1I2*, *GPBAR1*) diluted cDNA in combination with 2x master mix (Eurogentec, Maastricht, The Netherlands) in a total volume of 20 μl [21]. 18S mRNA levels were used as an internal control. Fluorescence was measured using the 7900HT Fast Real-Time System, and SDS 2.3 software (Applied Biosystems). Used primers and probes are listed in S1 Table.

### Purification of bile acids for GCDCA measurements

Preconditioned and/or challenged HepG2.rNtcp cells were washed twice with 1x Hank’s buffered salt solution (HBSS, life technologies) and harvested in 75% methanol (v/v) in demi water [22]. Samples were centrifuged for 10 min at 14,000x*g* at 4°C, supernatant was diluted 100-fold in 250 μl internal standard (IS) solution, containing a selection of deuterium-labelled (D_4_) bile acids. Samples were dried at 40°C under nitrogen and reconstituted in 200 μl 50% methanol (v/v) and filtered with a 0.2-μm centrifugal filter for 10 min at 3,000x*g* (VWR, Radnor, PA, USA).

### GCDCA measurement using UHPLC-MS/MS

For the quantitative determination of bile acids we used a Nexera X2 Ultra High Performance Liquid Chromatography system (SHIMADZU, Kyoto, Japan), coupled to a Sciex QTRAP 4500 MD triple quadrupole mass spectrometer (SCIEX, Framingham, MA, USA) (UHPLC-MS/MS). Bile acids were separated with a ACQUITY UPLC BEH C18 Column equipped with a ACQUITY UPLC BEH C18 VanGuard Pre-Column (Waters, Milford, MA, USA). Separation was achieved in 28 minutes using 10 mM ammonium acetate in 20% acetonitrile (mobile phase A) and 10 mM ammonium acetate in 80% acetonitrile (mobile phase B). All bile acids were detected in negative mode. The peak area was calculated for each experimental sample and related to the peak area of the D_4_-labeled bile acids in the IS.

### Statistical analysis

All results are reported as the mean of at least three independent experiments ± standard error of the mean (SEM). Data was considered normally distributed, unpaired analysis of variance (ANOVA), with Bonferroni post hoc testing was used to determine significant changes between experimental groups. A P-value of <0.05 was considered significant.

## Results

### Preconditioning with sub-toxic levels of GCDCA protect HepG2.rNtcp cells against GCDCA-induced apoptosis, whereas menadione or TNF-α preconditioning aggravates GCDCA-induced apoptosis

**Fig 1:**
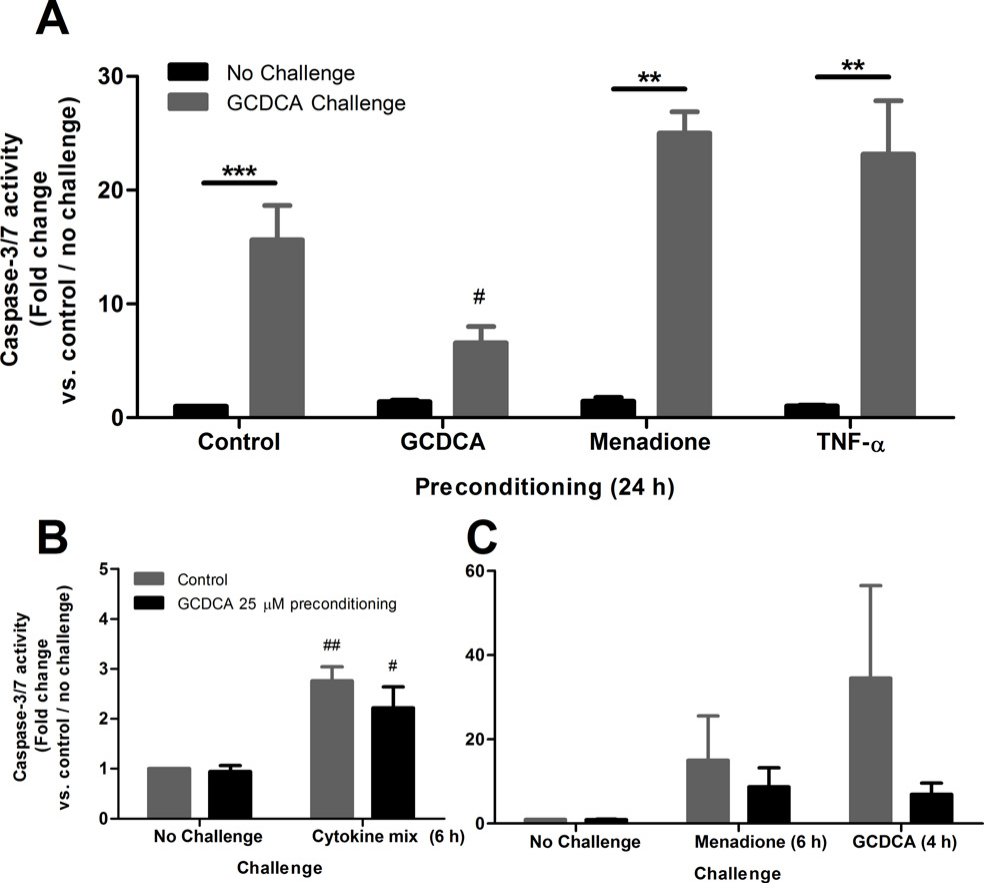
GCDCA preconditioning protects against GCDCA-induced apoptosis, whereas menadione and TNF-α preconditioning aggravate GCDCA-induced apoptosis. **(A)** HepG2.rNtcp cells were preconditioned with GCDCA (25 μM / 24 h), menadione (10 μM / 24 h) or TNF-α (10ng/ml / 24 hr) without challenge with GCDCA (Control, black bars) or challenged with the pro-apoptotic concentration (200 μM) GCDCA for 4 h (grey bars). **(B)** HepG2.rNtcp cells were preconditioned with 25 μM GCDCA for 24 h without challenge with GCDCA (Control, black bars) or challenged with pro-apoptotic concentrations of cytokine mix (see Material and Methods for details) or menadione (50 μM) for 6 h (grey bars). Caspase-3/7 activity was used as measurement of apoptotic cell death and is presented as fold change compared to control (no preconditioning and no GCDCA challenge). Control values were set to one. Experiments were performed n = 3–10 depending on the specific experiment and/or condition. Error bars represent the SEM. ** = P<0.01, *** = P<0.001, No challenge vs GCDCA challenge counterpart. # = P<0.05, # = P<0.01 compared to no preconditioning (24 h) / GCDCA challenge.

To determine which factors in cholestatic liver disease may aggravate or protect against bile acid-induced apoptosis, HepG2.rNtcp cells were preconditioned (for 24 h) with sub-apoptotic concentrations of GCDCA (25 μM), superoxide anion donor menadione (10 μM) [23] or pro-inflammatory cytokine TNF-α (10 ng/ml). After preconditioning, acute bile acid-toxicity was mimicked by challenging the cells with a pro-apoptotic concentration of 200 μM GCDCA for 4 h (**Fig 1A**). As expected, 24 h preconditioning with either GCDCA, menadione or TNF-α did not induce caspase-3/7 activity. Challenging HepG2.rNtcp cells with 200 μM GCDCA without preconditioning resulted in a strong (15.6-fold) induction in caspase-3/7 activity compared to control conditions, as reported earlier [5]. Preconditioning with 25 μM GCDCA for 24 h resulted in a pronounced 58% reduction in caspase-3/7 activity after the 200 μM GCDCA challenge compared to non-preconditioned cells. In contrast, preconditioning of HepG2.rNtcp cells with menadione or TNF-α strongly increased the caspase-3/7 activity after the 200 μM GCDCA challenge, compared to cells that did not underwent preconditioning.

In addition, we investigated whether preconditioning with GCDCA (25 μM, 24 h) protects against other hepatotoxic compounds. After preconditioning, cells were challenged with a cytokine mixture (CM) or 50 μM menadione for 6 hours and compared to the 4 h GCDCA challenge. GCDCA preconditioning did not significantly reduce CM- (**Fig 1B**) or menadione-induced apoptosis (**Fig 1C**). These data show that pre-exposure of HepG2.rNtcp cells to low concentrations of GCDCA protects cells against an apoptotic dose of this bile acid, but not against cytokine- or menadione-induced apoptosis. In addition, pre-exposure to tolerable amounts of reactive oxygen species or TNF-α aggravates bile acid-induced apoptosis.

### Preconditioning with GCDCA, TCA, and TUDCA protect against GCDCA-induced apoptosis

**Fig 2:**
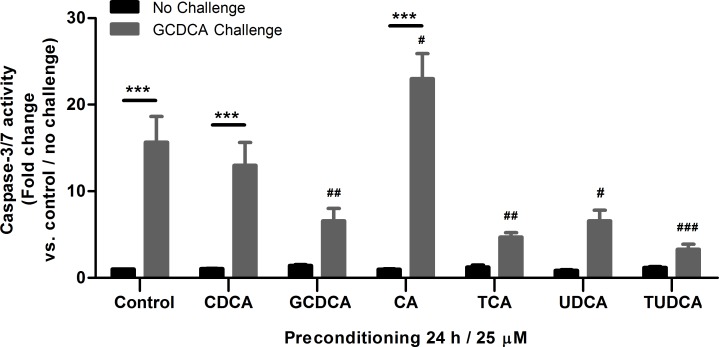
GCDCA, TCA and TUDCA preconditioning are most protective against GCDCA-induced apoptosis, compared to preconditioning with CDCA, CA or UDCA. HepG2.rNtcp cells were preconditioned with CDCA, GCDCA, CA, TCA, UDCA or TUDCA (25 μM / 24 h), without challenge with GCDCA (Control, black bars) or challenged with 200 μM GCDCA for 4 h (grey bars). Caspase-3/7 activity is shown as fold change compared to control (no preconditioning and no GCDCA challenge), control values were set as one. Experiment where performed n = 3–10 depending on the condition. Error bars represent the SEM. *** = P<0.001, No challenge versus GCDCA challenge counterpart. # = P<0.05, ## = P<0.01, ### = P<0.001, compared to no preconditioning (24 hr) / GCDCA challenge.

Next, we investigated whether preconditioning with other bile acids also provides protection to GCDCA-induced apoptosis. HepG2.rNtcp cells were preconditioned for 24 h with 25 μM of several unconjugated bile acids (CDCA, CA, and UDCA) and conjugated bile acids (GCDCA, TCA, and TUDCA), followed by the 200 μM GCDCA-apoptotic challenge for 4 h (**Fig 2**). Preconditioning with the conjugated bile acids GCDCA, TCA, TUDCA, and unconjugated UDCA all resulted in a reduction of caspase-3/7 activity after the GCDCA challenge compared to HepG2.rNtcp that were not preconditioned. The unconjugated bile acids CDCA and CA do not protect, while preconditioning with 25 μM CA even significantly enhances the caspase-3/7 activity induced by the GCDCA challenge. These data indicate that only conjugated bile acids (with exception of UDCA) are effective in protecting HepG2.rNtcp cells against GCDCA-induced apoptosis.

### Bile acid preconditioning does not lead to GCDCA-induced necrosis in HepG2.rNtcp cells

**Fig 3:**
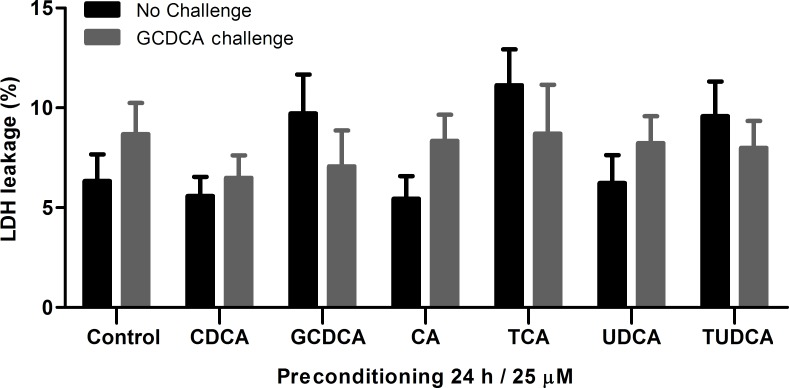
Bile acid preconditions does not induce a shift from apoptosis to necrosis when HepG2.rNtcp cells are exposed to 200 μM GCDCA. HepG2.rNtcp cells were preconditioned with CDCA, GCDCA, CA, TCA, UDCA or TUDCA (25 μM / 24 h), without challenge with GCDCA (Control, black bars) or challenged with the pro-apoptotic concentration (200 μM) GCDCA for 4 h (grey bars). LDH activity was measured in medium alone and in medium from digitonin-treated cells (100% leakage). LDH leakage is shown as percentage of total LDH. Experiments were performed in n = 3 for each condition. Error bars represent the SEM.

To investigate whether preconditioning with bile acids induces a shift from apoptotic to necrotic cell death, cellular leakage of lactate dehydrogenase (LDH) was determined after HepG2.rNtcp cells were exposed to the GCDCA challenge (**Fig 3**). The 200 μM GCDCA challenge for 4 h does not increase the LDH activity in the medium, indicating the absence of necrotic cell death in these conditions. Preconditioning with either unconjugated (CDCA, CA, UDCA) or conjugated (GCDCA, and TUDCA) did not cause a significant increase in LDH activity in the medium either before or after the GCDCA challenge. TCA preconditioning appeared to increase LDH release in the medium, although a large variation between experiments is observed. Thus, bile acid preconditioning does not induce a shift from apoptosis to necrosis when HepG2.rNtcp cells are exposed to 200 μM GCDCA and low dose conjugated bile acids truly provide protection to these cells.

### The protective effect of GCDCA preconditioning is time and concentration dependent

**Fig 4:**
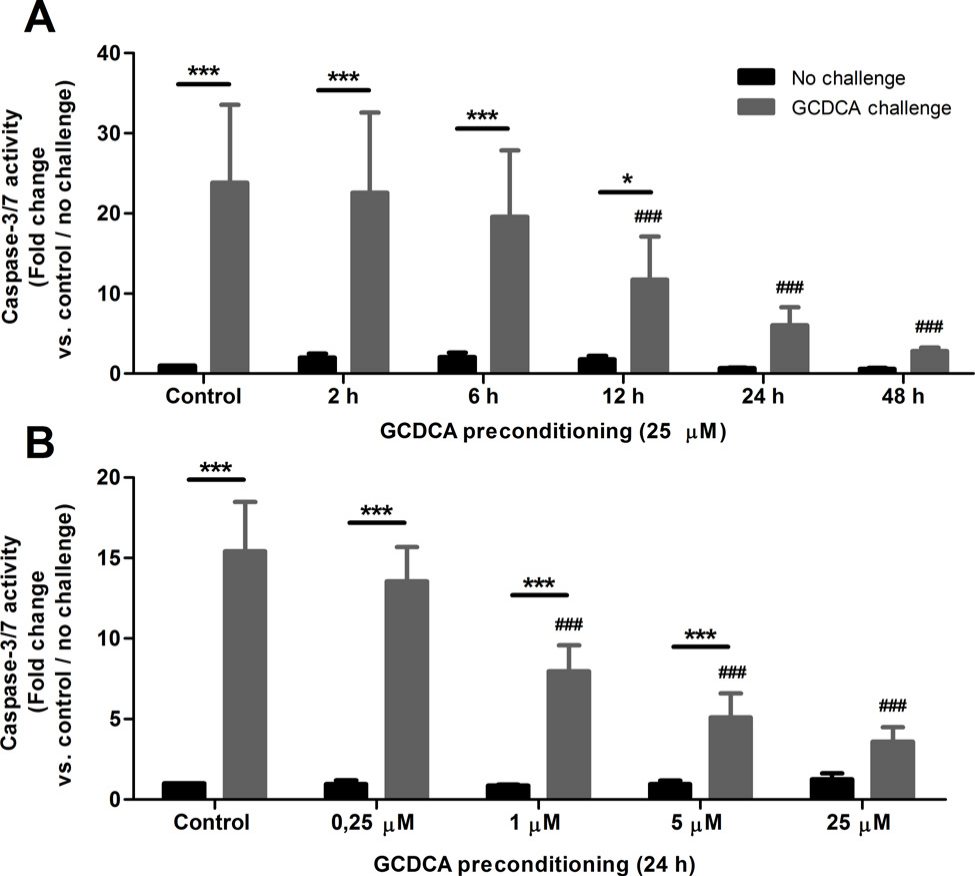
The protective effect of GCDCA preconditioning is time- and concentration-dependent. HepG2.rNtcp cells were preconditioned with GCDCA for different periods and in different concentrations. **(A)** Cells were preconditioned with 25 μM GCDCA for 2, 6, 12, 24, and 48 h, without challenge (control, black bars) or challenged 200 μM GCDCA for 4 h (grey bars). **(B)** Cells were preconditioned with 0.25, 1, 5, or 25 μM GCDCA for 24 h without challenge (control, black bars) or challenged 200 μM GCDCA for 4 h (grey bars). Caspase-3/7 activity is shown as fold change compared to control (no preconditioning and no GCDCA challenge), control values were set as one. Experiments were performed in n = 3 for each condition. Error bars represent the SEM. * = P<0.05, *** = P<0.001 No challenge versus GCDCA challenge counterpart. ### = P<0.001 compared to no preconditioning (24 h) / no GCDCA challenge.

To further characterize the cytoprotective mechanism(s) that are induced by low concentrations bile acids, we analysed the time- and GCDCA concentration-dependency of preconditioning. HepG2.rNtcp cells were pre-exposed to 25 μM GCDCA for 2, 6, 12, 24 and 48 h, followed by the 4 h 200 μM GCDCA challenge. The caspase-3/7 activity after the GCDCA challenge was significantly reduced when HepG2.rNtcp cells were preconditioned for at least 24 h, while a gradual decrease was observed in the intermediary time points (**Fig 4A**). Taking the standard 24 h preconditioning period, we next pre-exposed the HepG2.rNtcp cells to various concentrations of GCDCA (ranging from 0.25 to 25 μM) followed by the GCDCA challenge. A clear concentration dependent protective effect was observed for GCDCA in the preconditioning phase. Preconditioning with 1 μM GCDCA already leads to a significant (48.5%) reduction in the caspase-3/7 activity induced by the GCDCA challenge, which was further reduced at increasing GCDCA concentrations. Preconditioning with 25 μM GCDCA was most protective against the GCDCA challenge-induced apoptotic cell death, reducing the caspase-3/7 activity by 77% (**Fig 4B**).

### Downstream FXR target BSEP is induced by preconditioning with GCDCA and FXR agonist GW4064

**Fig 5:**
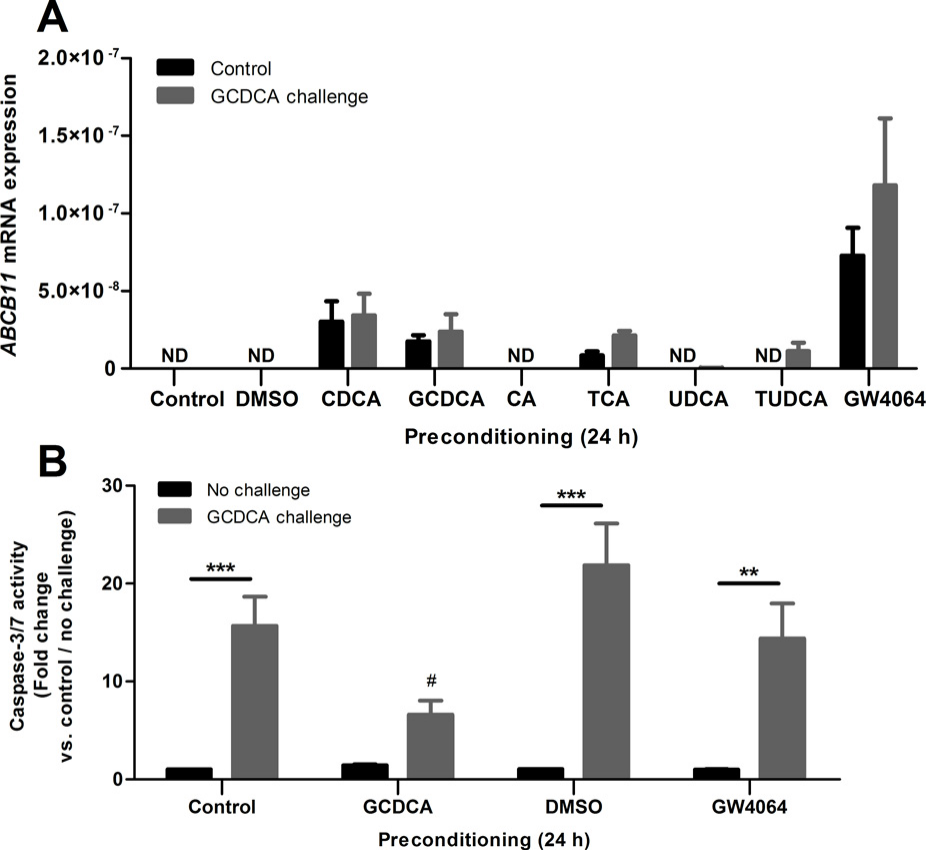
GCDCA, CDCA, and GW4064 preconditioning induces BSEP mRNA expression, and prevents GCDCA-induced apoptosis. HepG2.rNtcp cells were preconditioned with DMSO (1:1000; vehicle GW4064), the indicated bile acids (25 μM) or GW4064 (1 μM) for 24 hr without challenge (control, black bars) or challenged with 200 μM GCDCA for 4 hrs (grey bars). Error bars represent the SEM in all experiments. **(A)** mRNA expression was determined using qPCR, mRNA levels were corrected for 18S values are expressed in 2^-ΔCT^. Experiments are performed in n = 3–6. ND = Not detectable. **(B)** Cells were preconditioned with GCDCA or GW4064 (1 μM) for 24 h without challenge or challenged with 200 μM GCDCA for 4 h, caspase-3/7 activity is shown as fold-change compared to control (For GW4064 the control condition is DMSO preconditioning). Experiment was performed in n = 5–10. ** = P<0.01, *** = P<0.001, No challenge vs GCDCA challenge counterpart. ## = P<0.01, compared to no preconditioning (24 h) / GCDCA challenge.

Since the protective effect of GCDCA preconditioning was observed only after 24 h and at relatively low bile salt concentrations, we expected that transcriptional adaptations may be involved. Bile acids may act via FXR/RXR and induce, amongst others, transcription of the *ABCB11* gene, encoding the bile salt export pump (BSEP). Unconjugated CDCA is the prototypical ligand for FXR and clearly induced *ABCB11* mRNA levels in HepG2.rNtcp cells (**Fig 5A**). An identical induction of *ABCB11* levels was observed for GCDCA preconditioning, while unconjugated 25 μM CA did not enhance the *ABCB11* mRNA levels. A clear difference was observed in the protective effect of GCDCA and CDCA in the preconditioning phase (**Fig 2**). To discriminate between potential simultaneous protective and aggravating effects of CDCA, we used a synthetic (non-bile acid) FXR ligand for preconditioning, resulting in similar *ABCB11* levels. One (1) μM of GW4046 induced *ABCB11* expression to (at least) similar levels as 25 μM of CDCA or GCDCA after 24 h of exposure to HepG2.rNtcp cells (**Fig 5A**). The HepG2.rNtcp cells preconditioned with GCDCA, or GW4046 with or without the GCDCA challenge were next analysed for caspase-3/7 activity. Preconditioning with GCDCA resulted in a significant 58%, reduction of caspase-3/7 activity Preconditioning with GW4064 resulted in a reduction of 34% in caspase-3/7 activity, compared to DMSO (control) preconditioned cells (**Fig 5B**), indicating that FXR-mediated induction of *ABCB11*, and thereby enhanced bile salt efflux capacity of HepG2.rNtcp cells, may be part of the protective effect of GCDCA preconditioning.

### GCDCA preconditioning does not increase the GCDCA efflux of the cells after the GCDCA challenge

**Fig 6:**
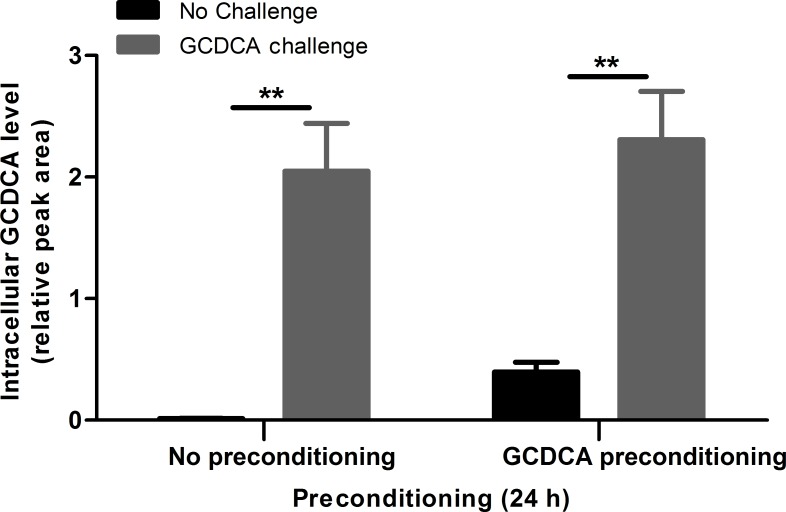
GCDCA preconditioning does not increase the efflux of GCDCA after the challenge. HepG2.rNtcp cells were preconditioned with GCDCA (25 μM / 24 h) without challenge with GCDCA (Control, black bars) or challenged with the pro-apoptotic concentration (200 μM) GCDCA for 4 h (grey bars). UHPLC-MS/MS was used to determine bile acid concentrations, intracellular GCDCA concentrations are shown as relative peak area. Error bars represent the SEM, experiment was performed in n = 3. ** = P<0.01, No challenge vs GCDCA challenge counterpart.

To investigate whether the increased *ABCB11* mRNA expression is an indication of an increased bile acid efflux of the cells we measured intracellular bile acid contents in our experimental model by using UHPLC-MS/MS (**Fig 6**). Cells were 24 h preconditioned with 25 μM GCDCA with or without subsequent GCDCA challenge (200 μM / 4h). In untreated cells, bile acid levels were below the lowest concentration of the standard curve (50 nM). Cells that were challenged for 4 h with 200 μM GCDCA showed strongly increased intracellular GCDCA levels, but those concentrations were not different between HepG2.rNtcp cells with or without GCDCA preconditioning. Thus, *ABCB11* levels in GCDCA–preconditioned HepG2.rNtcp cells were insufficient to lower intracellular GCDCA accumulation in challenged cells. FXR-independent mechanisms are therefore the main driving force of the protective effect of GCDCA preconditioning.

## Discussion

In this study, we show that GCDCA-induced apoptotic cell death in HepG2.rNtcp cells can be greatly reduced by preconditioning with a sub-apoptotic concentration of conjugated bile acids. In contrast, GCDCA-preconditioning does not suppress cytokine- or oxidative stress-induced apoptotic cell death. The protective effect of GCDCA- preconditioning is time- and concentration-dependent. Protection is observed after 12 h of preconditioning, with concentrations of GCDCA as low as 1 μM. In contrast to the effect of (conjugated) bile acids, the cytokine TNF-α and the ROS-inducer menadione aggravate GCDCA-induced apoptosis. GCDCA preconditioning induces *ABCB11* expression, theoretically enhancing GCDCA efflux. However, enhancement of *ABCB11* by the non-bile acid FXR agonist GW4064 shows only limited protection against GCDCA-induced apoptosis. Moreover, GCDCA-preconditioning did not suppress intracellular GCDCA accumulation after the apoptotic challenge. Hence, physical aspects of (conjugated) bile acids likely contribute most to the hormetic effect, possibly in conjunction with transcriptional regulation of factors other than *ABCB11*. UDCA is used in the treatment of several cholestatic liver diseases [9,10] and the protective effect of bile acids UDCA and TUDCA against GCDCA-induced apoptosis in *in vitro* models has been described before [5,24]. In this study, we found that with regard to preconditioning, the pro-apoptotic bile acids GCDCA and TCA are at least as potent in reducing GCDCA-induced apoptosis than UDCA and TUDCA. The protective effect of GCDCA has not been described before, but it is known that cells can adapt to cellular stress and thereby gain resistance to cytotoxic challenges. Previous studies have shown that ischemic preconditioning [25], preconditioning with hyperbaric hepatic hyperoxia [26], glutathione [27], or helium [28] is protective against ischemia/reperfusion injury after liver transplantation. Adaptation to cellular stress by preconditioning as observed in this study can be defined by the principles of hormesis. Hormesis refers to a response towards a compound that yields a stimulatory / favourable effect at low dosages, but an inhibitory / toxic effect at high concentrations [16]. Here, the HepG2.rNtcp cells adapt to low concentrations of bile acids in order to protect them against toxic levels of (the same) bile acids. In line with the concept of hormesis, GCDCA-preconditioning did not reduce cytokine- or oxidative stress-induced apoptotic cell death.

Moreover, we observed that preconditioning with the ROS-inducer menadione or the pro-inflammatory cytokine TNF-α aggravated GCDCA-induced apoptosis. In cholestatic liver disease, hepatocytes are exposed to inflammation (TNF-α production by Kupffer cells) and increased oxidative stress (e.g. mitochondrial dysfunction and/or infiltrating neutrophils). These detrimental conditions may contribute to, and even aggravate cholestatic liver damage [29,30]. It remains to be investigated whether inflammation and oxidative stress can reduce the therapeutic effect of bile acids like UDCA used in the treatment of cholestasis.

In general terms, we observed that preconditioning with conjugated bile acids protected against GCDCA-induced apoptosis, whereas unconjugated bile acids (with the exception of UDCA) did not show any protection. The rate and mechanism of transport into the hepatocyte differs between conjugated and unconjugated bile acids [31,32], which may be crucial for the protective effect of bile acid preconditioning.

The predominant mode of cell death during bile acid-toxicity is still debated. Depending on the species and experimental model used (*in vitro* or *in vivo*) apoptotic or necrotic cell death is observed. In most *in vitro* models (e.g. (transfected) hepatoma cell lines and primary hepatocytes) apoptosis is predominantly observed [11,33,34]. Yet, exposure to high concentrations of bile acids results primarily in necrotic cell death [35,36]. In *in vivo* models of cholestasis (e.g. experimental obstructive cholestasis; bile duct ligation; BDL), apoptosis is only observed to a limited extent and then mostly in the acute phase of cholestasis [11]. After the acute phase, hepatocytes appear to die mainly from necrosis [11,37]. In *in vivo* models the pathophysiology of bile acid toxicity is more complicated, due to inflammation and involvement of additional liver cell types. Previous research in rats exposed to bile duct ligation suggested that hepatocytes become resistant towards bile acid-induced apoptosis during progression of cholestasis and switch to necrosis [11]. This data together with other studies, indicate that bile acid-induced apoptosis is mainly observed in the acute phase of cholestasis, or exposure to low concentrations of bile acids (micromolar concentrations). In this study, no shift from apoptosis to necrosis was observed when hepatocytes were preconditioned with bile acids and subsequent exposed to the GCDCA challenge. This indicates that preconditioning with (conjugated) bile acids is truly protective for the HepG2.rNtcp cells and does not induce necrosis.

The protective effect of GCDCA preconditioning is both time- and concentration-dependent, with most pronounced protective effects after 24 h of preconditioning at a concentration of 25 μM. This suggests that transcriptional and/or physical adaptations of hepatocytes are involved in the protective effect of bile acid preconditioning. The late protective effect of preconditioning has been described as late preconditioning and depends mainly on increased expression of protective genes [38,39]. Protective pathways via NF-κB [11] and HO-1 induction [38] may be involved, however, this remains to be investigated.

A protective effect of GCDCA preconditioning was already observed at concentrations of 1 μM GCDCA, which is lower than the bile acid levels in serum of healthy humans [40]. Thus, hepatocytes in physiological conditions are always exposed to a basal level of bile acids. We speculate that because of this permanent exposure to bile acids, hepatocytes have a basal protection against bile acid-induced toxicity, suggesting an intrinsic hormetic response is present in hepatocytes *in vivo*. This might explain why in human cholestatic conditions very little apoptosis is observed.

We demonstrated that preconditioning with GCDCA (independent from the GCDCA challenge) induces BSEP (*ABCB11)* mRNA expression, while *NR1H4* mRNA levels (encoding FXR) were not affected (**S1 Fig**). Importantly, HepG2 cells express similar levels of FXR compared to human hepatocytes, with only minor differences in the relative amounts of the 4 isoforms [41]. Induction of BSEP might be one of the protective mechanisms of preconditioning as it exports GCDCA and thereby prevents the accumulation of cytotoxic concentrations of this bile acid compared to non-preconditioned cells. The importance of BSEP in cellular bile acid homeostasis is well documented. However, GCDCA-preconditioning did not prevent the sharp increase in intracellular GCDCA levels upon the 200 μM GCDCA challenge, strongly arguing against enhanced bile acid efflux through BSEP. In fact, this may not be surprizing as *ABCB11* mRNA levels are 500-fold lower in GCDCA-preconditioned HepG2.rNtcp cells compared to normal human hepatocytes (**S2 Fig**). In addition, FXR agonist (GW4064) preconditioning showed the strongest induction of BSEP expression, but did not protect against GCDCA-induced cell death to the same extend as GCDCA preconditioning. Moreover, GCDCA and CDCA lead to similar BSEP mRNA expression, where CDCA preconditioning did not lead to a significant protective effect, in contrast to GCDCA preconditioning, Taken together, these data point to mechanisms independent of bile acid transport that are responsible for the hormetic effect of GCDCA preconditioning. Expression of *NR1I2* (encoding PXR) was strongly decreased by both GCDCA-preconditioning and -challenge, whereas only the GCDCA challenge induced *GPBAR1* mRNA levels (encoding TGR5) (**S1 Fig**), which does not provide clues for involvement of these alternative bile acid sensors in the protective effect of GCDCA-preconditioning. Transcriptional pathways other than enhanced bile acid transport that may contribute to cytoprotection include 1) bile acid metabolism, 2) prevention of DNA damage and 3) shifting the balance of pro-apoptotic/pro-survival signalling, as has been described for cisplatin-mediated regulation of FXR targets [42]).The latter study showed that cisplatin enhanced FXR-mediated expression of *TCEA2*, encoding Transcription elongation factor A protein 2, and *KRT13*, encoding Keratin 13. These proteins have are involved in transcription-coupled DNA repair mechanisms and anti-apoptotic signaling, respectively. The potential role of such factors in the protective effect of GCDCA-preconditioning of HepG2.rNtcp cells awaits further analysis.

Though we do not exclude the involvement of transcriptional changes in the GCDCA-induced hormetic effect, it seems likely that physical changes to (cellular) membranes may also have a prominent role herein.

At high concentrations (micellar) bile acids damage membranes through their detergent action [43]. Moreover, bile acids are able to damage mitochondria, not so much through their detergent action, but rather by opening the mitochondrial permeability transition pore (MPTP) [44]. Opening of the MPTP results in depolarization of the mitochondria, release of cytochrome C, resulting in oxidative stress and activation of pro-apoptotic pathways eventually leading to cell death [44,45]. Preconditioning with sub-apoptotic concentrations of toxic bile acids might induce functional changes in cellular membranes, mitochondrial membranes and/or configuration of the MPTP providing protection against GCDCA-induced cell death. However, this hypothesis warrants further investigation.

Taken together, we conclude that HepG2.rNtcp cells are protected by a hormetic response against GCDCA-induced cell death by preconditioning with (conjugated) bile acids. The protective effect of preconditioning is not a result of FXR-controlled stimulation of BSEP-mediated GCDCA efflux. Additional mechanisms that contribute to the hormetic effect of bile acid preconditioning need further investigation.

## Supporting Information

S1 Fig Expression of different bile acid sensors upon GCDCA-preconditioning of HepG2.rNtcp cells with or without GCDCA-challenge. HepG2.rNtcp cells were preconditioned with GCDCA (25 μM) for 24 hr without challenge (control, black bars) or challenged with 200 μM GCDCA for 4 hrs (grey bars). mRNA expression of *NR1H4* (FXR), *NR1I2* (PXR) and *GPBAR1* (TGR5) was determined using qPCR and corrected for 18S.Values are expressed in 2^-ΔCT^ and error bars represent the SEM, experiment was performed in n = 3.
(TIF)

S2 Fig Comparison of *ABCB11* mRNA levels in HepG2.rNtcp cells and primary human hepatocytes. HepG2.rNtcp cells were preconditioned with GCDCA (25 μM) for 24 hr. Purified primary human hepatocytes were obtained from Tebu-Bio (Heerhugowaard, the Netherlands). mRNA expression of *ABCB11* (BSEP) was determined using qPCR and corrected for 18S. Values are expressed in 2^-ΔCT^ and error bars represent the SEM, experiment was performed in n = 3.
(TIF)

S1 Table Primers and probes used in this study. (PDF)
