# Supplementary figures and images for: Hormesis in Cholestatic Liver Disease; Preconditioning with Low Bile Acid Concentrations Protects against Bile Acid-Induced Toxicity
Source: PLoS One. 2016 Mar 7;11(3):e0149782. doi: 10.1371/journal.pone.0149782 (PMC4780766; doi:10.1371/journal.pone.0149782)

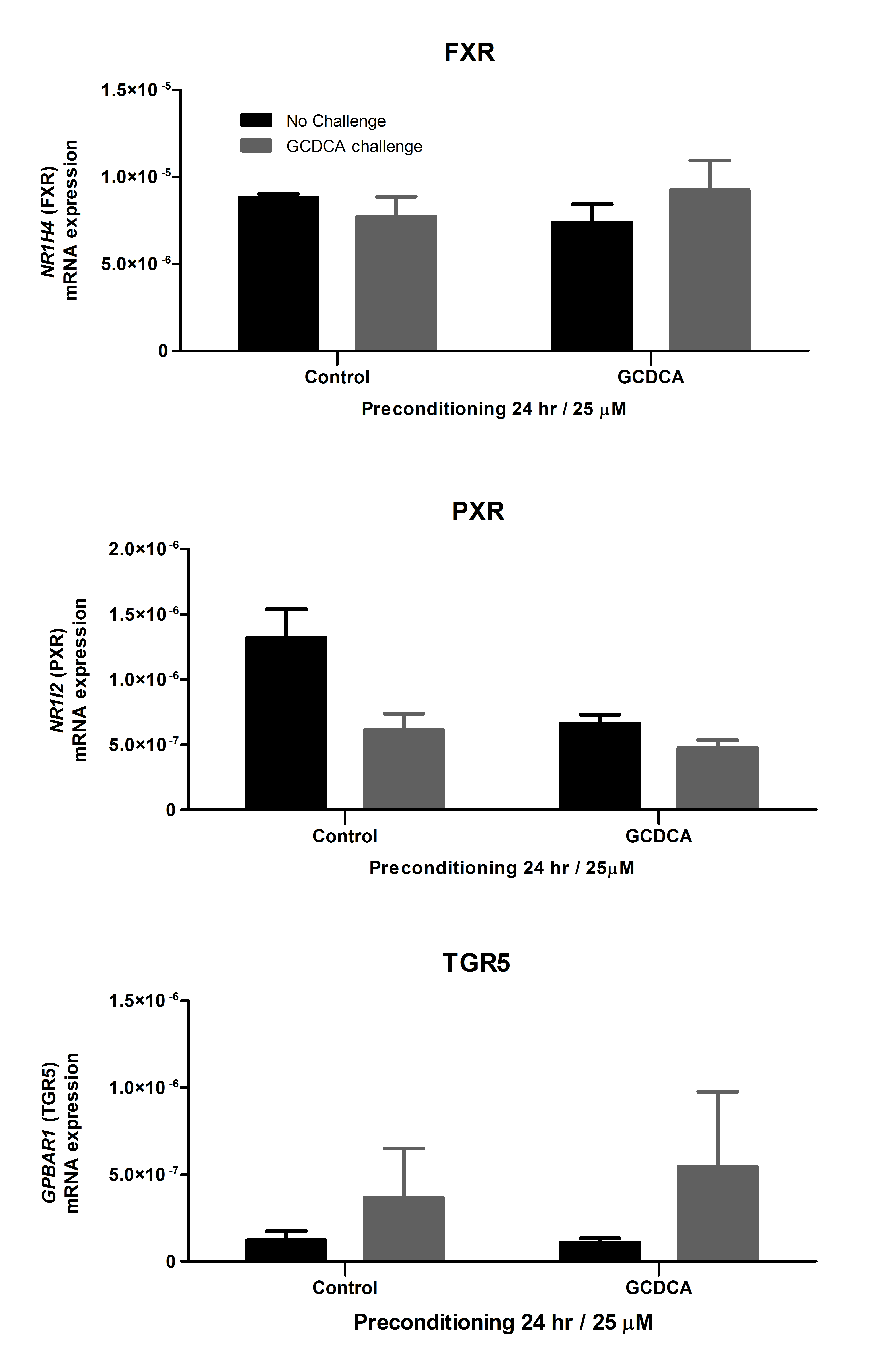

Supplement: S1 Fig — HepG2.rNtcp cells were preconditioned with GCDCA (25 μM) for 24 hr without challenge (control, black bars) or challenged with 200 μM GCDCA for 4 hrs (grey bars). mRNA expression of NR1H4 (FXR), NR1I2 (PXR) and GPBAR1 (TGR5) was determined using qPCR and corrected for 18S.Values are expressed in 2-ΔCT and error bars represent the SEM, experiment was performed in n = 3. (TIF) [file pone.0149782.s001.tif]

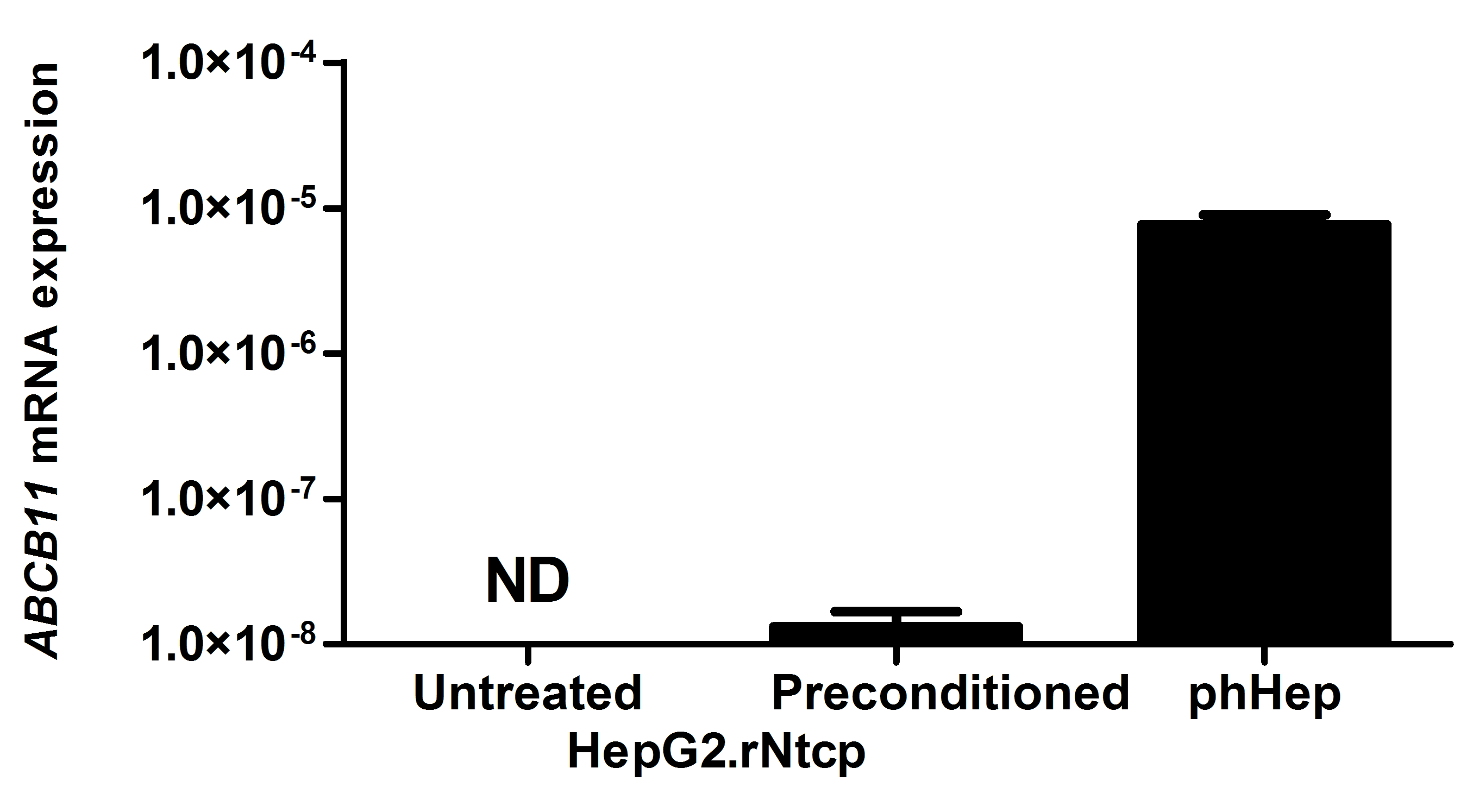

Supplement: S2 Fig — HepG2.rNtcp cells were preconditioned with GCDCA (25 μM) for 24 hr. Purified primary human hepatocytes were obtained from Tebu-Bio (Heerhugowaard, the Netherlands). mRNA expression of ABCB11 (BSEP) was determined using qPCR and corrected for 18S. Values are expressed in 2-ΔCT and error bars represent the SEM, experiment was performed in n = 3. (TIF) [file pone.0149782.s002.tif]
