# Supplementary material for: Hormesis in Cholestatic Liver Disease; Preconditioning with Low Bile Acid Concentrations Protects against Bile Acid-Induced Toxicity
Source: PLoS One. 2016 Mar 7;11(3):e0149782. doi: 10.1371/journal.pone.0149782 (PMC4780766; doi:10.1371/journal.pone.0149782)
Supplement: S1 Table — (PDF) [file pone.0149782.s003.pdf]

18S Forward primer	5'-CGG CTA CCA CAT CCA AGG A-3’
18S Reverse primer	5'-CCA ATT ACA GGG CCT CGA AA-3’
18S Probe	5'-CGC GCA AAT TAC CCA CTC CCG A-3’
BSEP Forward primer	5’-ACA TGC TTG CGA GGA CCT TTA-3’
BSEP Reverse primer	5’-GGA GGT TCG TGC ACC AGG TA-3’
BSEP Probe	5’-CCA TCC GGC AAC GCT CCA AGT CT-3’
FXR Forward primer	5’-ACC ACG CTG AGA TGC TGA TG-3’
FXR Reverse primer	5’-CAT CAC TGC ACG TCC CAG AT-3’
FXR Probe	5’-ACG ACC ACA AGT TTA CCC CAC TTC TCT GTG-3’
FXRα1+2 Forward primer	5’-TTC TGA AAA TTT ATT TGG TGT TTT AAC AGA-3’
FXRα1+2 Reverse primer	5’-TGG AAT AAT AGG ATG ACG AGG AAA TC-3’
FXRα1+2 Probe	5’-CAT TGC TGT ATT GCG AGT ATG GTT CCA CTT CC-3’
TGR5 Forward primer	5’-CGT CTA CTT GGC TCC CAA CTT C-3’
TGR5 Reverse primer	5’-GGC CTC AGG ACT GCC ATG TA-3’
TGR5 Probe	5’CTC TCC CTG CTT GCC AAC CTC TTG C-3’
PXR Forward primer	5’-AGC AAT TCG CCA TTA CTC TGA AGT-3’
PXR Reverse primer	5’-CTG GGT GTG CTG AGC ATT GA-3’
PXR Probe	5’-AGA TCA TGG CTA TGC GCA CCG AGC TCC-3’
